# Supplementary material for: Efficacy of the Simeox® Airway Clearance Technology in the Homecare Treatment of Children with Clinically Stable Cystic Fibrosis: A Randomized Controlled Trial
Source: Children (Basel). 2023 Jan 23;10(2):204. doi: 10.3390/children10020204 (PMC9955024; doi:10.3390/children10020204)
Supplement: Supplementary file 1 [file children-10-00204-s001.zip › children-2054946-supplementary.pdf]

Supplementary Material

Supplementary Materials and Methods

Ethical Considerations

According to the Polish Law, the study documents including informed consent form, and subsequent amendments were reviewed by an Independent Ethics Committee (IEC), the "KOMISJA BIOETYCZNA przy INSTYTUCIE MATKI I DZIECKA".

The regulatory authorization for conducting the study was obtained in accordance with applicable regulatory requirements. All approval documents were available before a subject was exposed to any trial-related procedure, including screening tests for eligibility.

The study was conducted in accordance with the ethical principles that have their origins in the Declaration of Helsinki (1964), as amended in Fortaleza (2013), in compliance with the approved Clinical Investigation Plan (CIP) and the rules governing medical devices as well as any local regulations.

Patient information and consent

Prior to study entry, all patients and their legally appointed and authorized representative were fully informed verbally and in writing about the nature and aim of the study. The Physician Investigator ensured that they all read and understood the patient information sheet. Patients and their legally appointed and authorized representative were also informed that their participation was voluntary and that they could withdraw from the study at any time without affecting their relationship with the Physician Investigator or access to any future treatment.

Before receiving any CIP procedures or treatments related to the trial, all the legally appointed and authorized representatives gave their written informed consent by signing and dating an EC approved printed information sheet and consent form explaining the potential benefits and possible side effects of the medical device Simeox®.

For each patient, two originals of the information sheet and consent forms were completed, dated and signed personally by the participant and by the Physician Investigator. One original was to be kept by the participant, the second original was kept by the Physician Investigator.

Confidentiality of data

All legal requirements regarding protection of personal data were adhered to. Patients in ascending order who satisfied the inclusion and exclusion criteria were assigned successive patient numbers in the order in which they were recruited in the clinical centre.

The anonymity of trial subjects was maintained. Throughout documentation and evaluation, the patients were identified on the data entry file and other documents by their identification number (pseudonym). Documents which identified the patient (e.g. the signed informed consent form) were maintained confidentially by the Physician Investigator. The patients were informed that all study findings were stored on computer and handled in the strictest confidentiality.

Any results and documents derived from the study were also regarded as confidential. The Physician Investigator and members of her research team were not allowed to disclose such information, except to authorized representatives of appropriate regulatory/health authorities, without prior written approval from the sponsor.

Collected data

The study included 3 visits for each group: Visit 0: Inclusion – Randomization, Visit 1: Group A and B: 1-month follow up, and Visit 2: Group A and B: 1-month follow up / end of study. Assessments at each visit are described in the *Table S1***Error! Reference source not found.**.

Medical history including presence of pancreatic insufficiency, sinus polyposis, liver disease, cystic fibrosis-related diabetes and chronic *Pseudomonas aeruginosa* infection, were obtained from hospital records. Patients performed spirometry, N_2MBW, IOS and BP at each of the study visits. At the same time, they completed the respiratory and physical functioning domain scores of the CFQ-R, as these are the most relevant domain for ACT assessment. The CFQ-R is a self-reported reliable and validated health-related quality of life measure that is specifically designed for patients with CF (1). At the end of treatment with Simeox patients were asked to complete treatment satisfaction questionnaires (comfort, pain, fatigue, ease of use, preference of intervention and recommendation to other patients).

Spirometry (Jaeger Vyntus IOS; CareFusion, Hochberg, Germany) was performed according to the American Thoracic Society/European Respiratory Society (ATS/ERS) criteria (2-4). Reference equations from the Global Lung Function Initiative (GLI) were used to calculate z-scores and percent predicted values for forced expiratory volume in 1 second (FEV_1), forced vital capacity (FVC) and other spirometry parameters.

N_2MBW was performed in order to calculate the lung clearance index (LCI), which is a lung function measure that can detect damage in small and large airways prior to changes in lung function determined using spirometry (5). N_2MBW tests were performed with the Exhalyzer-D (EcoMedics AG, Duernten, Switzerland**,** software version 3.2.0**)**. An N_2MBW test was considered successful if there were at least two or more technically acceptable tests in accordance with guidelines in the ATS/ERS consensus statement (6). All LCI results were expressed as the mean of at least two technically acceptable results obtained during one session; usually, the session included three or more tests. Due to its relevance, only LCI2.5 data were analyzed in the stat models.

In pursuance of the ATS/ERS criteria (3), BP was performed using Master Screen Body/Diff Jaeger (CareFusion, Hochberg, Germany). Spirometry and flow-volume curves were measured by whole-body plethysmography.

IOS is potentially affected by upper airway artifacts, in the form of swallows, vocal cord closures, coughs, incorrect positioning of the tongue, or mouth leaks. Therefore each test was coached by a technician. For all oscillometry tests, patients were breathing in a relaxed and stable manner, seated in an upright posture with the correct head position, cheek support, mouthpiece seal, and tongue position. Results of 3 technically acceptable measurements were used in the same mean resistance and reactance values (7).

Safety was determined based on the occurrence of adverse events and serious adverse events.

Table S1: Summary of the collected data during the study.

Collected Data	V0	V1	V2
Eligibility criteria	+		
Consent form	+		
Demographic data (gender, age)	+		
Clinical examination (height, weight)	+	+	+
Comorbidities	+		
Impulse Oscillometry System (IOS)	+	+	+
Clinical assessment (physical examination)	+	+	+
Assessment of Pulmonary Exacerbation	+	+	+
Plethysmography	+	+	+
Spirometry	+	+	+
N2MBW	+	+	+
AE/SAE	+	+	+
CFQ-R physical domain score	+	+	+
CFQ-R respiratory domain score	+	+	+
Concomitant medication	+	+	+
AE, SAE, deviations	+	+	+
Device deficiencies	+	+	+

Medical Device

Simeox® device allows to use different airway clearance techniques by completing conventional chest physiotherapy sessions with instrumental technique.

The electronic device consists of:

- a turbine generating depressions,
- a vibration generator,
- a microcontroller controlling the vibration frequency, and all the interfaces with the user,
- a data display interface and PC dialog for the analysis of results.

The Simeox® device is connected to a breathing system composed of:

- a personal or disposable mouthpiece or respirator,
- a protection filter for the single-use breathing chain,
- a flexible tube to be changed every seven uses,
- a machine protection filter to be changed every seven uses.

The Simeox® device consists of a turbine controlled by a microcontroller generating negative pressure pulses at a precise frequency of 12 Hz during the expiration (6 Hz for the last two expirations of the program). These successive negative pressure pulses are transmitted into the respiratory tract through the mouthpiece connected to a tube and a filter. During expiratory phases, the bronchial air of the patient is stimulated by Simeox®. Between each oscillation, the pulmonary pressure returns to atmospheric pressure. Using the properties of thixotropy of the respiratory mucus, this pressure variation is supposed to fluidify bronchial secretions and thus promote their mobilization by the mucociliary system and coughing mechanisms.

One session of Simeox® consists of about 20-40 respiratory cycles, and lasts about 20-30 min.

Each respiratory cycle includes:

- an inspiratory phase where the patient normally inspires, and
- an expiratory phase where the patient expires in the mouthpiece while activating the device by pushing on the remote control.

It is during the exhalation that the mucus is stimulated by oscillating successive negative pressure pulses and that it is liquefied. Once liquefied, the mucus can be easily expectorated by the patient.

The fluidification and drainage of the mucus proceeds in three phases:

1. Taking the mouthpiece in the mouth when expiring
2. Liquefaction of the mucus and mobilization under the effect of the vibratory stimulus: Thixotropy and air suction
3. Expectoration of liquefied mucus

Intended Use of the Medical Device

Simeox® from PHYSIO-ASSIST is a medical device intended to be used by adults and children over 8 years of age suffering from a chronic lung disease who have difficulty in clearing bronchial secretions. Simeox® medical device is a bronchial drainage assistance device for an efficient, less painful and autonomous bronchial clearance. By generating a series of negative pressure pulses of constant air volume, Simeox® mimics the vibrating cilia of the bronchial epithelium during normal relaxed expiration, this technique is efficient due to the significant decrease in the viscosity and increased mobility of the expectorated mucus.

This medical device is flexible and adapted to ambulatory use, Simeox® is intended for:

- Healthcare professionals (Doctors, Physiotherapists, to assist them in the decluttering phase (care establishment, physiotherapy practice)
- Patients at home to reduce the constraints related to their care, increase their autonomy to improve their quality of life.

Simeox® is indicated for airway secretion clearance in patients with respiratory diseases like Chronic Obstructive Pulmonary Diseases (COPD), Primary Ciliary Dyskinesia (PCD), Bronchiectasis, and Cystic Fibrosis (CF).

Interventions

The principle of Simeox airway clearance technology lays on rheological and thixotropic properties of mucus by modifying mucus viscosity and elasticity with a precise pneumatic vibratory stimulus (detailed description in Supplementary material). In Vitro and in vivo experimentations showed that Simeox device fluidifies and bring up sticky bronchial secretions and then helps them to be dislodged through coughing mechanism. The Simeox device spreads a vibratory pneumatic signal in the bronchial tree during relaxed exhalation by disseminating a succession of very short negative air pressure pulses of constant volume according to selected power at a 6hz and 12hz frequency. By this way, the signal mobilizes mucus and transport it from distal to proximal tract for its expectoration.

Simeox® device allows to use different airway clearance techniques by completing conventional chest physiotherapy sessions with instrumental technique.

Statistical analyses

Demographic and Baseline data

For the analysis of the demographic and baseline data, the Shapiro-Wilk test was used to determine the adequacy of normal distribution and then the Fisher-Snedecor test was used to verify homoscedasticity. If both distributions of the data followed a normal distribution and homoscedasticity was verified then Student's test was used. The Welsh test analyzed data in which both distributions were consistent with the normal distribution, but homoscedasticity was not verified. And if the data distributions did not follow a normal distribution then Wilcoxon Mann Whitney test was used (*Table S*).

Table S2: Statistical tests used to compare pulmonary function scores at baseline between Group A and Group B.

Variable	Shapiro-wilk test p-values (groups A/B)	Fisher-Snedecor test p-value (homoscedasticity)	Statistical test used to compare groups A and B
**Impulse Oscillometry**			
R 5Hz	**0.0047** * / 0.0660	-	Wilcoxon Mann Whitney
R5Hz-R20Hz	0.0541 / **≤ 0.001** *	-	Wilcoxon Mann Whitney
R 20Hz	**0.001** * / 0.262	-	Wilcoxon Mann Whitney
X 5Hz	0.2417 / 0.3754	**0.049****	Welch
AX	**≤ 0.001 * / ≤ 0.001 ***	**-**	Wilcoxon Mann Whitney
**Cystic Fibrosis questionnaire (CQF-R)**			
Physical score (patient)	0.0574 **/ 0.0037 ****	**-**	Wilcoxon Mann Whitney
Respiratory score (patient)	0.3311 **/ 0.0417 ***	**-**	Wilcoxon Mann Whitney
**Spirometry**			
Forced expiratory volume in one second (FEV1) z-score	0.8072 / **0.0068** *	-	Wilcoxon Mann Whitney
Forced vital capacity (FVC) z-score	0.4160 / 0.8874	**0.0238** *	Welch
Maximum Expiratory Flow at 25% (MEF25) z-score	0.6211 / 0.0721	0.3359	Student

Maximum Expiratory Flow at 50% (MEF50) z-score	0.9949 / **0.0068 ***	-	Wilcoxon Mann Whitney
Maximum Expiratory Flow at 75% (MEF75) z-score	0.9233 / 0.1728	0.2782	Student
**Lung clearance index**			
LCI 2.5	**0.0239 * / ≤ 0.001 *** **	-	Wilcoxon Mann Whitney
LCI 2.5 z-score	0.9987 / 0.2055	0.6636	Student
**Body plethysmography**			
Residual Volume (RV) z-score	**0.001 * / 0.0028 ***	-	Wilcoxon Mann Whitney
Total lung capacity (TLC) z-score	**0.0366** * / 0.0700	-	Wilcoxon Mann Whitney
RV/TLC z-score	**0.0053** * / 0.7950	-	Wilcoxon Mann Whitney
Functional residual capacity (FRC) z-score	**0.0378** * / 0.9818	-	Wilcoxon Mann Whitney
Airway resistance (Reff) z-score	**0.0499** * / 0.1073	-	Wilcoxon Mann Whitney
Airway resistance (sReff) z-score	**0.0012 * / 0.0015 ***	-	Wilcoxon Mann Whitney
Airway resistance (Rtot) z-score	**0.0227** * / **0.0112 ***	-	Wilcoxon Mann Whitney

Legends : P-value in bold with * indicates significance <0.05.

Comparison of treatments and comparisons of each treatment effect against baseline

Linear mixed-effects model analyses were performed using the following R packages: Rcmdr_2.7-1, RcmdrMisc_2.7-1, GGally_2.1.1, ggpubr_0.4.0, lmerTest_3.1-3, lme4_1.1-26 and car_3.0-10.

Details on the different models conducted to compare treatments and to compare each treatment against the baseline can be found in the *Table S and Table S.*

Table S3: Statistical models used to compare treatments effect (Simeox versus Conventional) on pulmonary function scores.

Variable	Type of analysis	Analyzed data **	Linear mixed-effects model
**Impulse Oscillometry**			
R 5Hz	Parametric model	All	R5HZ ~ TREATMENT + GROUP + R5HZ_BASELINE
R5Hz-R20Hz	Non-parametric test	All	-
R 20Hz	Parametric model	All	R20HZ ~ TREATMENT + GROUP + R20HZ_BASE-LINE
X 5Hz	Parametric model	only the first series of values at visit 2	X5HZ ~ TREATMENT + X5HZ_BASELINE
AX	Non-parametric test	All	-
**Cystic Fibrosis questionnaire (CQF-R)**			

Physical score (patient)	Parametric model	All	CFQRPPT ~ TREATMENT + GROUP + CFQRPPT_BASELINE
Respiratory score (patient)	Parametric model	All	CFQRRPT ~ TREATMENT + GROUP + CFQRRPT_BASELINE
**Spirometry**			
Forced expiratory volume in one second (FEV1) z-score	Non-parametric test	All	-
Forced vital capacity (FVC) z-score	Parametric model	All	FVCZS ~ TREATMENT + GROUP + FVCZS_BASELINE
Maximum Expiratory Flow at 25% (MEF25) z-score	Parametric model	All	MEF25ZS ~ TREATMENT + GROUP + MEF25ZS_BASELINE
Maximum Expiratory Flow at 50% (MEF50) z-score	Non-parametric test	All	-
Maximum Expiratory Flow at 75% (MEF75) z-score	Parametric model	All	Log(MEF75ZS) ~ TREATMENT + GROUP + MEF75ZS_BASELINE
**Lung clearance index**			
LCI 2.5	Parametric model	only the first series of values at visit 2	(LCI2.5 - LCI2.5_BASELINE) ~ TREATMENT + LCI2.5_BASELINE
LCI 2.5 z-score	Parametric model	only the first series of values at visit 2	LCI2.5ZS ~ TREATMENT + LCI2.5ZS_BASELINE
**Body plethysmography**			
Residual Volume (RV) z-score	Parametric model	All	RVZS ~ TREATMENT + GROUP + RVZS_BASELINE
Total lung capacity (TLC) z-score	Parametric model	All	TLCZS ~ TREATMENT + GROUP + TLCZS_BASELINE
RV/TLC z-score	Parametric model	only the first series of values at visit 2	RVTLCZS ~ TREATMENT + RVTLCZS_BASELINE
Functional residual capacity (FRC) z-score	Non-parametric test	All	-
Airway resistance (Reff) z-score	Non-parametric test	All	-
Airway resistance (sReff) z-score	Non-parametric test	All	-
Airway resistance (Rtot) z-score	Parametric model	All	Log(RTOTZS) ~ TREATMENT + GROUP + RTOTZS_BASELINE

Legends : ** Analyses were conducted on all data or only on the first series of values (visit 2) if there was a significant group effect or a significant interaction group x treatment effect when considering all data. Group contains two levels : A and B; and treatment includes two levels : Simeox and Conventional.

Comparison of treatments

The conditions of validity of each model were verified according to the independence of residuals, normality of residuals, and observed x fitted values. If the model diagnostics were satisfactory, estimated treatment effect (difference between Simeox® and conventional treatment) was given with a 95% confidence interval. When the parametric model was not satisfactory, transformations were implemented. But if the transformation was not efficient, non-parametric approach on ranked values was used. In this case, medians and first and third quartile for each treatment were given as well as the estimated treatment effect. If the group effect or the interaction between group and treatment was significant, models were re-built to compare treatments only on the first series of values (visit 2). More details on conducted models can be found in the supplementary data.

Table S4: Statistical models used to compare each treatment effect against baseline on pulmonary function scores.

Variable	Type of analysis	Analyzed Data **	Linear mixed-effects model
**Impulse Oscillometry**			
R 5Hz	Parametric model	All	R5HZ ~ TREATMENT + GROUP
R5Hz-R20Hz	Non-parametric test	All	-
R 20Hz	Parametric model	All	R20HZ ~ TREATMENT + GROUP
X 5Hz	Non-parametric test	All	-
AX	Non-parametric test	All	-
**Cystic Fibrosis questionnaire (CQF-R)**			
Physical score (patient)	Parametric model	All	CQFRPPT ~ TREATMENT + GROUP
Respiratory score (patient)	Parametric model	All	CQFRRPT ~ TREATMENT + GROUP
**Spirometry**			
Forced expiratory volume in one second (FEV1) z-score	Non-parametric test	only the first series of values at visit 2	-
Forced vital capacity (FVC) z-score	Parametric model	All	FVCZS ~ TREATMENT + GROUP
Maximum Expiratory Flow at 25% (MEF25) z-score	Parametric model	All	MEF25ZS ~ TREATMENT + GROUP
Maximum Expiratory Flow at 50% (MEF50) z-score	Parametric model	All	MEF75ZS ~ TREATMENT + GROUP
Maximum Expiratory Flow at 75% (MEF75) z-score	Non-parametric test	All	-
**Lung clearance index**			
LCI 2.5	Parametric model	only the first series of values at visit 2	LCI25 ~ TREATMENT
LCI 2.5 z-score	Non-parametric test	only the first series of values at visit 2	-
**Body plethysmography**			
Residual Volume (RV) z-score	Non-parametric test	only the first series of values at visit 2	-

Total lung capacity (TLC) z-score	Non-parametric test	All	-
RV/TLC z-score	Parametric model	All	RVTLCZS ~ TREATMENT + GROUP
Functional residual capacity (FRC) z-score	Parametric model	All	FRCZS ~ TREATMENT + GROUP
Airway resistance (Reff) z-score	Non-parametric test	All	-
Airway resistance (sReff) z-score	Parametric model	All	SREFFZS ~ TREATMENT + GROUP
Airway resistance (Rtot) z-score	Non-parametric test	All	-

Legends : ** Analyses were conducted on all data or only on the first series of values (visit 2) if there was a significant group effect or a significant interaction group x treatment effect when considering all data. Group contains two levels : A and B; Treatment includes three levels : Simeox, Conventional and Baseline.

Supplementary Results

Baseline pulmonary function scores

Table S5: Baseline pulmonary function scores.

Pulmonary function tests	All patients n = 40 Mean ± SD	Group A n = 20 Mean ± SD	Group B n = 20 Mean ± SD	p-value
**Impulse Oscillometry**				
R 5Hz	0.48 ± 0.16	0.49 ± 0.17	0.47 ± 0.14	0.8710
R 5- R 20Hz	0.08 ± 0.08	0.09 ± 0.08	0.07 ± 0.07	0.3492
R 20Hz	0.40 ± 0.11	0.40 ± 0.11	0.40 ± 0.10	0.9030
X 5Hz	-0.17 ± 0.07	-0.19 ± 0.08	-0.16 ± 0.05	0.2539
AX	0.80 ± 0.81	0.95 ± 0.96	0.66 ± 0.60	0.3167
**Cystic Fibrosis questionnaire (CQF-R)**				
Physical score (patient)	85.31 ± 13.52	82.43 ± 13.85	88.19 ± 12.88	0.1505
Respiratory score (patient)	80.42 ± 12.84	78.61 ± 13.67	82.22 ± 12.04	0.3620
**Spirometry**				
Forced expiratory volume in one second (FEV1) z-score	-0.75 ± 1.46	-1.35 ± 1.58	-0.16 ± 1.07	**0.0067 ***
Forced vital capacity (FVC) z-score	-0.24 ± 1.20	-0.78 ± 1.32	0.30 ± 0.77	**0.0035 ***
Maximum Expiratory Flow at 25% (MEF25) z-score	-1.08 ± 1.57	-1.58 ± 1.66	-0.58 ± 1.32	**0.0423 ***
Maximum Expiratory Flow at 50% (MEF50) z-score	-0.21 ± 1.78	-0.50 ± 2.14	0.08 ± 1.32	0.2184
Maximum Expiratory Flow at 75% (MEF75) z-score	-0.48 ± 1.76	-0.69 ± 1.98	-0.27 ± 1.54	0.4591

**Lung clearance index**				
LCI 2.5	10.67 ± 3.68	12.16 ± 4.09	9.19 ± 2.55	**0.0041 ***
LCI 2.5 z-score	8.26 ± 4.86	10.46 ± 4.59	6.06 ± 4.15	**0.0029 ***
**Body plethysmography**				
Residual Volume (RV) z-score	0.14 ± 3.31	0.88 ± 3.69	-0.61±2.79	**0.0123 ***
Total lung capacity (TLC) z-score	-0.35 ± 2.01	-0.49 ± 2.17	-0.23 ± 1.88	0.7572
RV/TLC z-score	1.10 ± 2.43	2.19 ± 2.47	0.02 ± 1.89	**< 0.001 ***
Functional residual capacity (FRC) z-score	0.55 ± 2.55	1.01 ± 2.92	0.10 ± 2.09	0.1333
Airway resistance (Reff) z-score	1.59 ± 2.65	1.98 ± 3.00	1.20 ± 2.27	0.6205
Airway resistance (sReff) z-score	2.78 ± 3.60	3.45 ± 4.16	2.10±2.90	0.2110
Airway resistance (Rtot) z-score	2.98 ± 2.66	3.38 ± 2.93	2.59 ± 2.36	0.3792

Legends: Forced Expiratory Volume in 1 second (FEV1), Forced Vital Capacity (FVC), Maximal Expiratory Flow (MEF), lung clearance index (LCI2.5), Functional residual capacity (FRC), Airway resistance (Rtot, kPa/(l/s)), Airway resistance (sReff, kPa*s), Airway resistance (Reff, kPa/(l/s)), Functional residual capacity (pleth), Residual Volume (RV), Total Lung Capacity (TLC), Central lung resistance (R20hz), Peripheral lung resistance (R5-20hz), Peripheral lung reactance (X5hz), Central lung reactance (X20hz), Area of reactance (AX), Resonant frequency (Fres).

The ITT population filled a satisfactory questionnaire at the end of the study (Table S). The Questionnaires included 15 questions on the utilization and satisfaction. The patients had multiple response options; for satisfaction questions the options were: Definitively yes, Probably yes, Definitively not, Probably not.

Interestingly, 95.0% of patients considered that the drainage with Simeox® was not causing discomfort or pain (“Definitively not” and “Probably not” responses). In addition, 92.5% of patients considered that it was easily relax to exhale (“Definitively yes” and “Probably yes” responses). As per the fatigue parameter of the questionnaire, 77.5% of patients considered that drainage with Simeox® did not cause fatigue (“Definitively not” and “Probably not” responses), and 100% of patients considered that Simeox® is easy to handle (“Very easy” and “Easy” responses).

57.5% of patients considered that it took only 1 to 2 drainage sessions to learn how to drain properly with Simeox®, where interestingly, 100% of patients could do the drainage completely independently (without the help of a physiotherapist). In line with these results, 72.5% of patients preferred drainage with Simeox® over the existing drainage forms (“Definitively yes” and “Probably yes” responses), and 95% of patients would recommend Simeox® to other patients (“Definitively yes” and “Probably yes” responses).

Table S6: Satisfactory questionnaire.

**Satisfactory questionnaire**	**All patients N = 40**
**1. Was the drainage causing discomfort or pain?**	
Definitively not, n (%)	24 (60.0)
Probably not, n (%)	14 (35.0)
Probably yes, n (%)	2 (5.0)

Satisfactory questionnaire	All patients<br>N = 40
Definitely yes, n (%)	0 (0)
**2. Could you easily relax as you exhale?**	
Definitively yes, n (%)	18 (45.0)
Probably yes, n (%)	19 (47.5)
Probably not, n (%)	3 (7.5)
Definitely not, n (%)	0 (0)
**3. Did drainage with Simeox cause fatigue?**	
Definitively not, n (%)	12 (30.0)
Probably not, n (%)	19 (47.5)
Probably yes, n (%)	7 (17.5)
Definitely yes, n (%)	2 (5.0)
**4. How much sputum did you cough up?**	
A lot, n (%)	0 (0)
Much, n (%)	13 (32.5)
Insignificant amount, n (%)	12 (30.0)
No discharge, n (%)	15 (37.5)
**5. What was the color of the expectorant discharge?**	
Clear, n (%)	9 (36.0)
Light green, n (%)	13 (52.0)
Dark green, n (%)	3 (12.0)
Brown with an admixture of blood, n (%)	0 (0)
**6. Is Simeox easy to handle?**	
Very easy, n (%)	25 (62.5)
Easy, n (%)	15 (37.5)
Hard, n (%)	0 (0)
Very difficult, n (%)	0 (0)
**7. How many drainage sessions did it take you to learn to drain properly with Simeox?**	
About 1-2, n (%)	23 (57.5)
About 3-4, n (%)	13 (32.5)
About 5-6, n (%)	4 (10.0)
About 7-8, n (%)	0 (0)
**8. Could you do the drainage completely independently (without the help of a physiotherapist)?**	
Definitively yes, n (%)	28 (70.0)
Probably yes, n (%)	12 (30.0)
Probably not, n (%)	0 (0)
Definitely not, n (%)	0 (0)

Satisfactory questionnaire	All patients<br>N = 40
**9. Do you prefer drainage with Simeox over the existing drainage forms?**	
Definitively yes, n (%)	10 (25.0)
Probably yes, n (%)	19 (47.5)
Probably not, n (%)	10 (25.0)
Definitely not, n (%)	1 (2.5)
**10. Would you like to use Simeox at home?**	
Definitively yes, n (%)	13 (32.5)
Probably yes, n (%)	18 (45.0)
Probably not, n (%)	8 (20.0)
Definitely not, n (%)	1 (2.5)
**11. Have you used a nose clip?**	
Always, n (%)	9 (22.5)
Often, n (%)	3 (7.5)
Rarely, n (%)	10 (25.0)
Never, n (%)	18 (45.0)
**12. Would you recommend Simeox to other patients?**	
Definitively yes, n (%)	15 (37.5)
Probably yes, n (%)	23 (57.5)
Probably not, n (%)	2 (5.0)
Definitely not, n (%)	0 (0)
**13. Duration of drainage**	
10-15 min, n (%)	5 (12.5)
15-20 min, n (%)	25 (62.5)
20-30 min, n (%)	7 (17.5)
>30 min, n (%)	3 (7.5)
**14. Number of exhalations in cycles**	
x6, n (%)	3 (7.5)
x8, n (%)	19 (47.5)
x10, n (%)	18 (45.0)
**15. Simeox power setting**	
25%, n (%)	10 (25.0)
50%, n (%)	25 (62.5)
75%, n (%)	5 (12.5)
100%, n (%)	0 (0)

Statistical analyses were carried out on the following populations:

- **ITT population** is defined as all enrolled and randomized patients during inclusion period and who have been treated with the SIMEOX medical device;
- **PP population** is defined as included patients from the ITT population without any major deviation from the protocol.

As shown in the descriptive data of the ITT population there was only one declared **Adverse event** in one patient from the Simeox® group (No adverse events were declared in the patients from the group without Simeox®) (Table S**Error! Reference source not found.**). The adverse event was a mild, not serious, not related to the medical device, and not treated hemoptysis episode that was declared in a patient from Group A (Table S).

Table S7: Adverse Events.

**Adverse events**	**ITT population n_{ITT} = 40**		
	All patients *N = 40*	*With Simeox* *n = 20*	*Without Simeox* *n = 20*
$n_{patients\ with\ at\ least\ one\ adverse\ event}$	1	1	0
$n_{included\ patients}$	40	40	40
$rate_{adverse\ events}$ (%)	2.5	2.5	0
$CI_{95\%}(rate_{adverse\ events})$ (%)	[0.1 ; 14.7]	[0.1 ; 14.7]	[0 ; 10.9]

Table S8: Adverse events listing.

Patient file ID	Patient code	ITT population	PP population	Sequence group	Event description	Event severity	Event intensity	Relationship with the medical device	Action(s) taken	Evolution
6	**6 - A**	Yes	Yes	Group A	Hemoptysis episode	Not serious	Mild	Not related	No treatment	-

Table S9: Pulmonary exacerbations.

**Pulmonary exacerbations**	**ITT population n_{ITT} = 40**		
	All patients *N = 40*	*With Simeox* *n = 20*	*Without Simeox* *n = 20*
**Pulmonary exacerbations**			
$n_{patients\ with\ at\ least\ one\ pulmonary\ exacerbation}$	0	0	0
$n_{included\ patients}$	40	40	40
$rate_{pulmonary\ exacerbations}$ (%)	0	0	0
$CI_{95\%}(rate_{pulmonary\ exacerbations})$ (%)	[0 ; 10.9]	[0 ; 10.9]	[0 ; 10.9]

Supplementary Discussion

Patient Population Discussion

In the current study the sample followed a simple randomization; the best part of simple randomization is that it minimizes any bias by eliminating predictability. Furthermore, each subject can maintain complete randomness and independence with regard to the treatment administered (8).

This method is easy to understand and apply, but it cannot prevent the imbalances in the sample size or prognostic factors that are likely to occur as the number of subjects participating in the study decreases. In a study involving a total of 40 subjects in two groups, if 20 subjects are allocated to each group, the power is 80% (9). If the total number of subjects is 40, the probability of the imbalance is 52.7%, but this decreases to 15.7% for 200 subjects and 4.6% for 400 subjects (9).

One can discuss that the likelihood of imbalance in trials with a small number of subjects is high when using simple randomization (10). However the current study used the cross-over study design providing the advantages of reducing the variability in outcome measures from outside confounders.

Discussion of Randomization

There was a significant difference, when comparing FEV1 between Group A and Group B, of 22.5% (P=0.0259; Student test). This difference in the baseline values did not impact the estimated treatment effect Simeox® – CPT; we could not tell if the significant difference in baseline results of both groups had a further impact on the FEV1 for the mixed model analysis as this parameter was not further included in the mixed model analysis.

In addition, a significant difference, when comparing FEV1 (predicted) between Group A and Group B, of 15% was found (P=0.0076; Wilcoxon Mann Whitney test). Indeed this has no impact on the further analysis of the results in the mixed model as the difference is below 20%. Although a non-parametric test (only with the first series of values at visit 2) was used in the mixed model for FEV1 (Predicted), and there was a significant difference between group A and B at the baseline, this difference is inferior to 20% thus the **non-significant result seen in the mixed model** is credible.

Finally, a significant difference, when comparing FEV1 (z score) between Group A and Group B, of 157% was found. A non-parametric test (only with the first series of values at visit 2) was used in the mixed model of FEV (z score), and there was a significant difference between group A and B superior to 20%. Thus the non-significant results of the mixed model when comparing both Simeox® to baseline and CPT to baseline are of limited interpretation.

Another parameter was shown to present significant differences in the randomization results. A significant result was found, when comparing the FVC between Group A and Group B, of 18.6%.). Indeed this has no impact on the further analysis of the results as the difference is below 20%. Moreover, the FVC results were not included in the mixed model analysis.

Similar results were found when comparing the FVC (predicted) results and a significant difference of 12.93% was found. Although a non-parametric test (only with the first series of values at visit 2) was used in the mixed model, and there was a significant difference between group A and B at the baseline, this difference is inferior to 20% thus the **non-significant result seen in the mixed model** is credible. Furthermore, significant results of 138% were found when comparing the FVC (z score) when comparing Group A to Group B. A non-parametric test (only with the first series of values at visit 2) was used in the mixed model of FVC (z score), and there was a significant difference between group A and B superior to 20%. Thus the non-significant results of the mixed model when comparing both Simeox® to baseline and CPT to baseline are of limited interpretation.

Another parameter was shown to present significant differences in the randomization results. A significant result was found, when comparing the MEF25 (raw data) between Group A and Group B, of 47%. This difference in the baseline values did not impact the estimated treatment effect Simeox® – CPT as shown in table 34. We could not tell if the

significant difference in baseline results of both groups had a further impact on the MEF25 (raw value) for the mixed model analysis as this parameter was not further included in the mixed model analysis.

In addition, a significant difference, when comparing MEF25 (predicted) between Group A and Group B, of 35.8% was found (P=0.0438; Student test). Indeed this has an impact on the further analysis of the results in the mixed model as the difference is above 20%. However, a parametric test was used in the mixed model for MEF25 (Predicted) to compare the whole data avoiding the imbalance seen in the randomization, and the **non-significant result seen in the mixed model** is credible. Finally, a significant difference, when comparing MEF25 (z score) between Group A and Group B, of 63.3% was found. A parametric test of the whole data was further used in the mixed model of MEF25 (z score), and there was a non-significant difference. Thus the non-significant results of the mixed model when comparing both Simeox® to baseline and CPT to baseline are credible.

The analysis of the Residual volume (%) when comparing group A and Group B demonstrated a significant change of -25.57 % (p=0.0123; Wilcoxon Mann Whitney). We could not tell if the significant difference in baseline results of both groups had a further impact on the RV (%) for the mixed model analysis as this parameter was not further included in the mixed model analysis. However, a significant difference, when comparing RV (z score) between Group A and Group B, of -169% was found. A non-parametric test (only with the first series of values at visit 2) was used in the mixed model, and there was a significant difference between group A and B superior to 20% thus a limited interpretation of the non-significant results of the mixed model is concluded for this parameter.

The analysis of the RV/LTC when comparing group A and Group B demonstrated a significant change of -30.9 % (p≤0.001; Wilcoxon Mann Whitney). We could not tell if the significant difference in baseline results of both groups had a further impact on the RV (%) for the mixed model analysis as this parameter was not further included in the mixed model analysis. Similarly The analysis of the RV/LTC (%) when comparing group A and Group B demonstrated a significant change of -25.8 % (p=0.0011; Wilcoxon Mann Whitney). We could not tell if the significant difference in baseline results of both groups had a further impact on the RV (%) for the mixed model analysis as this parameter was not further included in the mixed model analysis. Finally, The analysis of the RV/LTC (z score) when comparing group A and Group B demonstrated a significant change of -99 % (p≤0.001; Wilcoxon Mann Whitney). Then a parametric test to compare the whole data avoiding the imbalance seen in the randomization was used in the mixed model for RV/LTC (z score), and the non-significant results seen in the mixed model are credible.

The analysis of the LCI2.5 (raw data) when comparing group A and Group B demonstrated a significant change of -24.4 % (p=0.0041; Wilcoxon Mann Whitney). A Non-parametric test (only with the first series of values at visit 2) was used in the mixed model for this parameter, and there was a significant difference between group A and B superior to 20% thus the non-significant results of Baseline-Simeox® and significant results of Baseline-CPT are of limited interpretation. In addition, although the analysis of the LCI2.5 (z score) when comparing group A and Group B demonstrated a significant change of -42.1 % (p=0.0029; Student), a parametric test was used in the mixed model for LCI2.5 (z score) to compare the whole data avoiding the imbalance seen in the randomization, and the **non-significant results of Baseline-Simeox and the significant results of Baseline-CPT seen in the mixed model** are credible.

Finally, the significant difference in randomization results for the LCI5, LCI5 (z score), VT(2), VT(2) (z score), M1M0, M1M0 (z score), M2M0, M2M0 (z score), may have an impact on the mixed model analysis of these outcome measures. However, these measure were not included in the mixed model analysis.

References

1. Sands D, Borawska-Kowalczyk U. Polska adaptacja Kwestionariusza Jakości Życia przeznaczonego dla dzieci i dorosłych chorych na mukowiscydozę oraz ich rodziców (CFQ-R). Pediatria Polska. 2009;84(2):165-72.
2. Beydon N, Davis SD, Lombardi E, Allen JL, Arets HG, Aurora P, et al. An official American Thoracic Society/European Respiratory Society statement: pulmonary function testing in preschool children. Am J Respir Crit Care Med. 2007;175(12):1304-45.
3. Miller MR, Hankinson J, Brusasco V, Burgos F, Casaburi R, Coates A, et al. Standardisation of spirometry. Eur Respir J. 2005;26(2):319-38.

4. Graham BL, Steenbruggen I, Miller MR, Barjaktarevic IZ, Cooper BG, Hall GL, et al. Standardization of Spirometry 2019 Update. An Official American Thoracic Society and European Respiratory Society Technical Statement. Am J Respir Crit Care Med. 2019;200(8):e70-e88.
5. Horsley A. Lung clearance index in the assessment of airways disease. Respir Med. 2009;103(6):793-9.
6. Robinson PD, Latzin P, Verbanck S, Hall GL, Horsley A, Gappa M, et al. Consensus statement for inert gas washout measurement using multiple- and single- breath tests. Eur Respir J. 2013;41(3):507-22.
7. King GG, Bates J, Berger KI, Calverley P, de Melo PL, Dellacà RL, et al. Technical standards for respiratory oscillometry. Eur Respir J. 2020;55(2).
8. Altman DG, Bland JM. Statistics notes. Treatment allocation in controlled trials: why randomise? BMJ. 1999;318(7192):1209.
9. Lim CY, In J. Randomization in clinical studies. Korean J Anesthesiol. 2019;72(3):221-32.
10. Schulz KF. Randomized controlled trials. Clin Obstet Gynecol. 1998;41(2):245-56.
